# Do Mini–Mental State Examination and Montreal Cognitive Assessment predict high‐cost health care users? A competing risks analysis in The Irish Longitudinal Study on Ageing

**DOI:** 10.1002/gps.5766

**Published:** 2022-06-15

**Authors:** Peter May, Céline De Looze, Joanne Feeney, Soraya Matthews, Rose Anne Kenny, Charles Normand

**Affiliations:** ^1^ The Irish Longitudinal Study on Ageing School of Medicine Trinity College Dublin Dublin Ireland; ^2^ Centre for Health Policy and Management Trinity College Dublin Dublin Ireland; ^3^ Cicely Saunders Institute of Palliative Care Policy and Rehabilitation King's College London London UK

**Keywords:** Alzheimer's disease, cognition, dementia, health care costs, utilisation

## Abstract

**Objectives:**

Policymakers want to better identify in advance the 10% of people who account for approximately 75% of health care costs. We evaluated how well Mini–Mental State Examination (MMSE) and Montreal Cognitive Assessment (MoCA) predicted high costs in Ireland.

**Methods/Design:**

We used five waves from The Irish Longitudinal Study on Ageing, a biennial population‐representative survey of people aged 50+ (2010–2018). We used competing risks analysis where our outcome of interest was “high costs” (top 10% at any wave) and the competing outcome was dying or loss to follow‐up without first having the high‐cost outcome. Our binary predictors of interest were a ‘low score’ (bottom 10% in the sample) in MMSE (≤25 pts) and MoCA (≤19 pts) at baseline, and we calculated sub‐hazard ratios after controlling for sociodemographic, clinical and functional factors.

**Results:**

Of 5856 participants, 1427 (24%) had the ‘high cost’ outcome; 1463 (25%) had a competing outcome; and 2966 (51%) completed eight years of follow‐up without either outcome. In multivariable regressions a low MoCA score was associated with high costs (SHR: 1.38 (95% CI: 1.2–1.6) but a low MMSE score was not. Low MoCA score at baseline had a higher true positive rate (40%) than did low MMSE score (35%). The scores had similar association with exit from the study.

**Conclusions:**

MoCA had superior predictive accuracy for high costs than MMSE but the two scores identify somewhat different types of high‐cost user. Combining the approaches may improve efforts to identify in advance high‐cost users.

## Key points

- Policymakers want to better identify in advance the 10% of people who account for approximately 75% of health care costs, since many of those costs may be modifiable.
- Cognitive function and cognitive decline are often cited as associated with formal health care costs but relationships are not well understood.
- In multivariable regressions, baseline Montreal Cognitive Assessment (MoCA) score was predictive of membership of the “high cost” group but baseline Mini–Mental State Examination (MMSE) was not.
- The scores identify different types of high‐cost user. Identification strategies may benefit from combining the two scores.

## 1 INTRODUCTION

### 1.1 Background

Health care costs are typically concentrated among a small minority of service users.^1^, ^2^, ^3^ While health care use is associated with serious disease, functional impairment and proximity to death, high costs often reflect poor‐quality and low‐value care including unplanned hospital admissions and high‐intensity care inconsistent with preferences.^4^ The quality and value of care to this group might be achieved by better anticipation of complex needs and provision of new supports.^4^, ^5^ Improved ex ante identification of the target patient population is an essential first step to providing such supports but challenging in practice.^5^, ^6^ This is sometimes called the “denominator challenge”: any sub‐group with high health care costs will be drawn from a large, heterogeneous population with high prevalence of serious conditions and limitations, and so specific prospective identification of at‐risk cases is extremely difficult.^6^

Alzheimer's disease and related dementias (ADRD) are shaping this challenge in important ways. Compared to other serious life‐limiting conditions, the longer trajectory from diagnosis to death means that people stay high‐need users for longer.^7^ Care needs result in higher provision of ongoing supports in community and institutional settings, as well as higher reliance on unpaid family carers.^8^ Higher prevalence of ADRD as populations age has enormous economic consequences for societies worldwide.^9^, ^10^

### 1.2 Rationale and aim

Multiple tools to predict future cognitive decline and ADRD risk exist. Little is known about how useful these tools are in improving advance identification of the highest‐cost group of interest to policy makers. We evaluated two well‐known measures of cognitive function–the Mini Mental State Examination (MMSE) and Montreal Cognitive Assessment (MoCA)–for their predictive usefulness in identifying high‐cost service users.

Our specific research questions were:

1. Do low MMSE and MoCA scores at baseline subsequently predict high health care costs among older people in Ireland during 8 years of longitudinal follow‐up?
2. Does one score perform notably better than the other in prediction?
3. Does the comparison usefully inform efforts to address the “denominator challenge” into the future?

## 2 METHODS

### 2.1 Study design

This study is a secondary analysis of The Irish Longitudinal Study on Ageing (TILDA). The Irish Longitudinal Study on Ageing is a prospective nationally representative study of older adults living in the community in the Republic of Ireland (ROI). Details of the TILDA survey design, participant sampling and data collection methods are available elsewhere.^11^ Briefly, the study collects data on a wide range of topics including health and health care use, economic and family circumstances, and life history. At Wave 1 (2009–2011), each participant completed a computer‐assisted personal interviews (CAPI) and self‐completion questionnaires (SCQ), and a comprehensive health assessment by a trained nurse.^12^, ^13^, ^14^ Both MMSE and MoCA at baseline were collected at the health assessment. Subsequent waves are biennial (Wave 2 in 2012, Wave 3 in 2014, etc). CAPI and SCQ follow‐up occurs at each Wave; health assessments were conducted again at Wave 3. In the event of a participant death, TILDA approaches a family member or close friend to complete a voluntary interview on end‐of‐life experience; full details including ethical procedures have been provided previously.^3^ This study uses data up to and including Wave 5 (2018).

### 2.2 Setting

Ireland is an island in north‐western Europe comprising two jurisdictions: ROI, which is an independent country, and Northern Ireland, which is a part of the United Kingdom. The Irish Longitudinal Study on Ageing is conducted within the ROI (ROI) only. ROI has a population of approximately 4.9 million people, with a relatively young age distribution for a high‐income country.^15^ The healthcare system has mixed public and private provision. A means‐tested medical card confers free primary care and hospital visits, and also caps co‐payments for prescriptions.^16^ Those without a medical card pay full primary care costs out of pocket as well as capped co‐payments for hospital care and prescriptions. Over half of people aged 50+ have voluntary health insurance to access private hospital care in the context of lengthy waiting lists for planned care.^17^ Compared to similar countries the ROI health system has unusually high acute hospital bed occupancy and relatively low primary and community care provision.^18^

### 2.3 Variables

#### 2.3.1 Predictors

Our primary predictors of interest were MMSE and MoCA at Wave 1. An MMSE cut‐point of 24 points has been suggested previously to identify normal function but in practice a range of points are used.^19^ We created a binary variable in MMSE where the participant had a value of 1 with 25 MMSE points or fewer. Previous research has found that this has good sensitivity and specificity.^20^ The developers of MoCA advocated a cut point of 26 or lower to indicate mild cognitive impairment (MCI),^21^ but this has been shown to have low specificity.^22^, ^23^ A range of alternative cut‐offs on MoCA have been used depending on the cohort. The two tests have differing purposes–MMSE was derived with respect to detecting dementia, MoCA to detect MCI–and as such the distributions of these scores differ within a given sample.^24^ To ensure that observed differences in comparing MMSE and MoCA were not due to sample size, we defined a binary MoCA variable at the same percentile as we created the binary MMSE variable.

To identify additional predictors we drew on Andersen's model of predisposing, enabling, need characteristics and prior utilisation^16^; and prior cost modelling using TILDA data.^3^ The full list of predictors is provided in Table 1.

**TABLE 1:** Descriptive data: characteristics of the analytic sample at baseline (Wave 1; *N* = 5856)

Variable		All	Lowest 10% both MoCA & MMSE	Lowest 10% MoCA, not MMSE	Lowest 10% MMSE, not MoCA	Lowest 10% neither MoCA nor MMSE
		N = 5856	n = 318	n = 218	n = 216	n = 5104
Age	*Years*	63.1 (9.3)	72.1 (10.5)	69.9 (10.1)	66.8 (10.1)	62.12 (8.7)
Sex	*Male*	2687 (45.9)	136 (42.8)	97 (44.5)	112 (51.9)	2342 (45.9)
Education^b^						
	*Primary/none*	1523 (26.0)	223 (70.1)	126 (58.1)	117 (54.4)	1057 (20.7)
	*Secondary*	2409 (41.2)	83 (26.1)	73 (33.6)	75 (34.9)	2178 (42.7)
	*Third/Higher*	1922 (32.8)	12 (3.8)	18 (8.3)	23 (10.7)	1869 (36.6)
Living situation						
	*Alone*	1171 (20.0)	121 (38.1)	70 (32.1)	51 (23.6)	929 (18.2)
	*With spouse*	2384 (40.7)	110 (34.6)	78 (35.8)	92 (42.6)	2104 (41.2)
	*With others*	2301 (39.3)	87 (27.3)	70 (32.1)	73 (33.8)	2071 (40.6)
Area						
	*Dublin*	1522 (26.0)	44. (13.8)	29 (13.3)	43 (19.9)	1406 (27.6)
	*Other urban*	1607 (27.4)	87 (27.4)	64 (29.4)	59 (27.3)	1397 (27.4)
	*Rural*	2727 (46.6)	187 (58.8)	125 (57.3)	114 (52.8)	2301 (45.1)
Insurance^c^						
	*Private insurance*	2646 (45.2)	31 (9.8)	30 (13.8)	40 (18.5)	2545 (49.9)
	*Medical card*	1549 (26.7)	208 (65.4)	111 (50.9)	96 (44.4)	1134 (22.2)
	*Dual cover*	1061 (18.1)	61 (19.1)	60 (27.5)	52 (24.1)	888 (17.4)
	*Neither*	596 (10.2)	18 (5.7)	17 (7.8)	28 (13.0)	533 (10.5)
ADLs^d^	*Total /6*	0.14 (0.55)	0.55 (1.2)	0.31 (0.88)	0.18 (0.58)	0.11 (0.45)
IADLs^a^	*Total /6*	0.12 (0.56)	0.63 (1.3)	0.31 (0.86)	0.26 (0.90)	0.07 (0.40)
Cancer dx^c^	*Yes*	361 (6.2)	24 (7.6)	16 (7.3)	13 (6.0)	308 (6.0)
Heart dx	*Yes*	561 (9.6)	57 (17.9)	33 (15.2)	33 (15.3)	438 (8.6)
ADRD dx^c^	*Yes*	8 (0.1)	4 (1.3)	1 (0.5)	1 (0.5)	2 (<0.5)
Multimorbidity	*Yes*	1466 (25.0)	115 (36.2)	79 (36.4)	64 (29.6)	1208 (23.7)
Hospital stay	*Yes*	1234 (21.1)	84 (26.5)	62 (28.4)	53 (24.5)	1035 (20.2)

*Note*: For binary and categorical variables: N (%). For continuous and count variables: Mean (St D).

Abbreviations: (I)ADL: (Instrumental) Activities of Daily Living. Dx = self‐reported diagnosis. Cancer = excluding skin cancer. Heart = at least one of congestive heart failure, heart attack, stroke. ADRD: Alzheimer's disease and related dementias. Multimorbidity = two or more of the following conditions angina, heart attack, CHF, stroke, cardiac arrhythmia, hypertension, diabetes, chronic lung disease, cancer, Parkinson's, psychiatric problems including depression or anxiety, alcohol or drug abuse, dementia, stomach ulcers, cirrhosis or serious liver damage, thyroid problems, kidney damage. Hospital stay: inpatient visit or emergency department (ED) admission in the last 12 months.

Prevalence of missing data:

^a^ n = 1

^b^ n = 2

^c^ n = 3

^d^ n = 4.

#### 2.3.2 Outcome

Our primary outcome of interest was binary: was the participant in the top 10% of costs in the first five waves of TILDA? We first established the 90^th^ percentile of costs in pooled data across all five waves, and then identified if participant costs exceeded this value in at least one of Wave 2, Wave 3, Wave 4 or Wave 5. The Irish Longitudinal Study on Ageing asks about formal utilisation of public and private services across 21 domains (e.g. hospital, general practitioner, nursing home and residential care, home care, allied health care). Costs were calculated by combining self‐reported frequency of service use with unit costs of each service. Units costs were calculated previously adapting the UK's Personal Social Services Research Unit methodology to the Irish context^25^, ^26^ and standardised to 2018, the final year of data collection, using the consumer price index.^27^ If an end‐of‐life interview was conducted for a participant who died in a given wave, we calculated costs for that person in that wave from the end‐of‐life interview. We categorised the outcome as binary because we were interested specifically in predicting the high‐cost class, and not in the association between coefficients and the full outcome distribution.

Our secondary outcome of interest was mortality. All registered deaths in the ROI are recorded with the General Register Office (GRO). The Irish Longitudinal Study on Ageing data between Wave 1 and Wave 5 are linked to the GRO data to March 2018, in a procedure detailed elsewhere.^28^ In addition to this, TILDA may become aware of participant deaths after being notified by a family member or after the TILDA team approach for an interview. To account for this, we have a mortality file providing full coverage of participant death dates within Ireland during the time period (Wave 1 to Wave 5), via the GRO, and additional non‐comprehensive coverage on deaths outside the State (from family members).

### 2.4 Statistical methods

In descriptive analyses we examined the overall sample on baseline predictors, and we stratified the sample by MMSE and MoCA scores. In presenting our outcome data we reported the distribution of costs in the sample at each wave, and we calculated the proportion of total sample costs that are accounted for by the top 10% of the distribution.

In our main analyses we used regression to analyse the association between our predictors, low MMSE and low MoCA scores, and our outcome, high costs at any wave. We conducted unadjusted bivariate regressions and multivariable regressions adjusting for factors in Table 1. At each wave, TILDA participants may die or drop out of the study. Since mortality and dropping out are not independent of our predictors, simply treating these outcomes as missing data increases the risk of bias.^29^, ^30^, ^31^ We treated these outcomes as competing risks; that is, events that potentially prevent occurrence of the primary outcome of interest but which should not be treated as missing in analysis.^32^ At wave 2, if a participant was in the top 10% of costs then they were deemed to have the outcome of interest, if the participant had died or did not participate they were deemed to have the competing risk, and if they had neither then they were retained to examine outcomes at wave 3, and so on. Thus we estimate associations between predictors and outcome, after taking account of any participants who had died or dropped out and could not achieve the outcome. As such our analyses adjust for participants' mortality rather than allocating the deceased zero costs or dropping them from the analysis.^33^

### 2.5 Bias

While TILDA was representative of the community‐dwelling population aged 50+ at baseline, MMSE and MoCA data were collected only for the sub‐sample who attended the health assessment. While all participants were invited to attend the health assessment, approximately 30% did not do so. This group is therefore missing from the analysis and not at random. In the Appendix we present summary statistics for our sample alongside the Wave 1 participants who did not complete MoCA and MMSE. Missingness on baseline variables at Wave 1 and on specific categories of health care use at later waves among the analytic sample was very low. In primary analysis we imputed missing variables using the age‐adjusted median, and we checked robustness of results to this strategy by rerunning without those observations that had missing data. In sensitivity analyses we checked our results to different cut‐offs using equipercentile methodology.^24^

## 3 RESULTS

### 3.1 Descriptive data

The characteristics of the sample are summarised in Table 1. There were 5856 participants completing the health assessment, including the MMSE and MoCA questionnaires; this was 70% of all Wave 1 participants (Appendix). Missingness was no higher than four people (<0.1%) on any Table 1 variable. The average age of the sample was 63.1 years and 46% were male. For level of education, a quarter had achieved primary (26.0%), 41.2% had achieved secondary and a third (32.8%) had achieved tertiary. Almost half of respondents (46.6%) lived in a rural area, with the remainder spread evenly between the capital city Dublin (26.0%) and other urban areas (27.4%). A similar number of people had a medical card (44.6%) or private insurance without a medical card (45.2%), and only 10.2% had neither coverage. Mean activities of daily living requiring help was 0.14 and mean instrumental activities of daily living was 0.12. Six per cent of the sample had a cancer diagnosis, 9.6% had a serious heart condition, 0.1% had an ADRD diagnosis and 25% had at least two serious chronic conditions.

**FIGURE 1:**
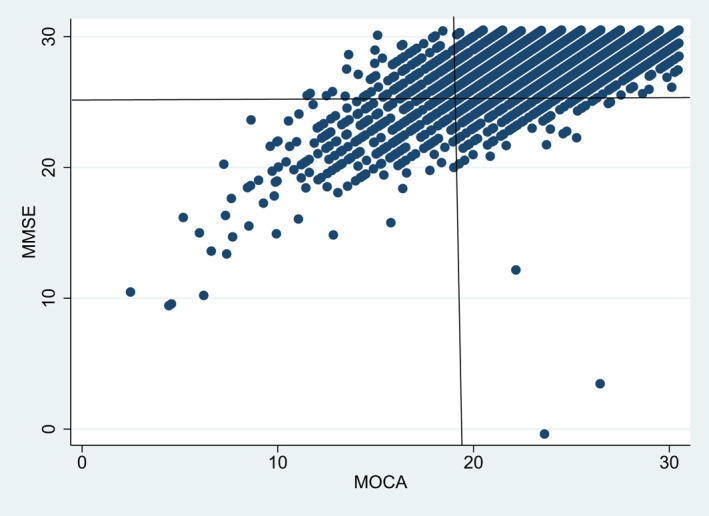
Scatterplot of MoCA and MMSE scores at baseline for the analytic sample (*N* = 5,856)

Baseline scores for the MMSE and MoCA scores are presented in Figure 1. Our MMSE cut‐point of 25 MMSE points or fewer was the lowest 10% of respondents at Wave 1. We therefore cut MoCA at the 10^th^ percentile also; this binary MoCA predictor had a value of 1 for participants with 19 MoCA points or fewer.

Baseline characteristics for sub‐samples stratified by MMSE and MoCA scores are also presented in Table 1. Of the analytic sample, 5104 (87%) had neither low MMSE nor low MoCA scores (i.e. upper‐right quadrant in Figure 1); 318 (5%) had both low MMSE and low MoCA scores (lower‐left quadrant); 218 (4%) had low MoCA but high MMSE scores (lower‐right quadrant); and 216 (4%) had low MMSE but high MoCA scores (upper‐left quadrant). The group with low scores on both indices were older and other characteristics reflected associations with age in older populations: more likely to be female, lower educational achievement, to live alone, to live in a rural area, to have a medical card, to report functional limitations and to have diagnosis of serious chronic diseases.

### 3.2 Outcome data

The health care costs across five waves are presented in Table 2, alongside numbers of the sample to achieve our primary outcome of interest (top 10% of costs) or a competing risk (death or dropping out of the study). The 90^th^ percentile of costs across all five waves was €9,718, and any participant whose costs exceeded this point in a year were classed as achieving the high‐cost outcome. In our analytic sample, 1427 (24%) had the “high cost” outcome during 8‐year follow‐up; 1463 (25%) had the competing outcome; and 2966 (51%) completed five waves without either outcome. Mean costs in the sample were steady over time, from €3018 at Wave 2 to €2918 at Wave 5. At each Wave, the 10% highest‐cost participants accounted for 72%–75% of total sample health care costs.

**TABLE 2:** Outcome data: outcomes by wave and costs across five waves (€)

	Outcomes by wave				Cost distribution		
	Sample (N = )	Competing risk: died or lost to follow‐up (n = )	Primary outcome: High‐cost outcome (n = )	Neither outcome (n = )	Mean	Min‐10%‐25%‐50%‐75%‐90%‐max	% Of total costs accounted for by 10% most costly
Wave 2	5856	401	477	4978	3018	0‐48‐96‐274‐728‐9434‐279,128	75%
Wave 3	4978	400	412	4166	3198	0‐48‐96‐280‐827‐9396‐266,965	74%
Wave 4	4166	331	277	3558	2619	0‐48‐96‐274‐775‐8382‐192,144	72%
Wave 5	3558	331	261	2966	2918	0‐48‐96‐288‐793‐8605‐198,047	73%

*Note*: High‐cost outcome = yes if costs for that wave were greater that €9718 (=90^th^ percentile for all costs pooled across five waves).

### 3.3 Main results

The regression results are presented in Table 3. Confidence intervals (95% CI) are presented and statistically significant results (*p* < 0.05) highlighted bold. In the unadjusted models, both low MMSE (SHR: 1.63 (CI: 1.4–1.9)) and low MoCA (SHR: 1.99 (CI: 1.7–2.3)) scores were significantly associated with high health care costs. In the adjusted models, MoCA (SHR: 1.38 (CI: 1.2–1.6)) exhibited a statistically significant association but MMSE did not. Estimated associations between other predictors and outcome were highly consistent across multivariable regressions. Age, male sex, living alone, living in Dublin, dual medical card and insurance cover, functional limitations, multimorbidity and hospital stay were all significantly positively correlated with membership of the high‐cost group in both the MMSE and MoCA regression.

**TABLE 3:** Multivariate regression results: competing‐risks analysis

Variable		Unadjusted models		Adjusted models	
MMSE	*Lowest 10%*	1.63 (1.4 to 1.9)***		1.15 (1.0–1.4)	
MoCA	*Lowest 10%*		1.99 (1.7 to 2.3)***		1.38 (1.2–1.6)***
Age	*Years*			1.02 (1.0–1.0)***	1.01 (1.0‐1.0)***
Sex	*Male*			1.13 (1.0–1.3)*	1.14 (1.0–1.3)*
Education	*Secondary*			1.11 (1.0–1.1)	1.13 (1.0–1.2)
	*Third/Higher*			1.01 (0.9–1.2)	1.04 (0.9–1.2)
Living situation	*With spouse*			0.74 (0.7–0.8)***	0.75 (0.7–0.9)***
	*With others*			0.79 (0.7–0.9)**	0.79 (0.7–0.9)**
Area	*Other urban*			0.82 (0.7–0.9)**	0.81 (0.7–0.9)**
	*Rural*			0.81 (0.7–0.9)**	0.80 (0.7–0.9)***
Insurance	*Insurance*			0.97 (0.8–1.2)	0.97 (0.8–1.2)
	*Medical card*			1.18 (0.9–1.5)	1.17 (0.9–1.4)
	*Dual cover*			1.24 (1.0–1.6)	1.25 (1.0–1.6)*
ADLs	*1*			1.26 (1.1–1.5)*	1.26 (1.0–1.5)*
	*2*			1.41 (1.0–1.9)*	1.39 (1.0–1.9)*
	*3+*			1.06 (0.7–1.5)	1.01 (0.7–1.5)
IADLs	*1*			1.53 (1.2–1.9)***	1.51 (1.2–1.9)***
	*2+*			1.79 (1.4–2.3)***	1.75 (1.4–2.3)***
Cancer dx	*Yes*			1.09 (0.9–1.3)	1.10 (0.9–1.3)
Heart dx	*Yes*			1.19 (1.0–1.4)*	1.20 (1.0–1.4)**
ADRD dx	*Yes*			0.71 (0.3–1.7)	0.69 (0.3–1.6)
Multimorbidity	*Yes*			1.42 (1.2–1.6)***	1.42 (1.2–1.6)***
Hospital stay	*Yes*			1.35 (1.2–1.5)***	1.35 (1.2–1.5)***

*Note*: Sub‐hazard ratios (95% confidence interval): association with membership of the high‐cost class in any wave. *p < 0.05; **p < 0.01; ***p < 0.001. For variables Legend, see Table 1.

Summary statistics of health care costs at exit among those in the highest‐cost group are presented in Table 4. Mean costs were highest (€33,548) among those with both low MMSE and low MoCA scores at baseline. Those with low MMSE but not low MoCA had costs approximately 10% lower (€30,231). Those with low MoCA but not low MMSE had considerably lower costs (€24,060) than those two groups, comparable to the majority of participants who had neither low MoCA nor low MMSE at baseline (€23,127). Among the 536 participants who had a low MoCA score at baseline, 217 had the high‐cost outcome subsequently–a true positive rate of 40%. Among the 534 participants who had a low MMSE score at baseline, 186 had the high‐cost outcome subsequently–a true positive rate of 35%.

**TABLE 4:** Mean costs (€) at exit among those who had the high‐cost outcome (n = 1427), by Mini–Mental State Examination (MMSE) and Montreal Cognitive Assessment (MoCA) baseline scores

		Low MMSE at baseline		
		*No*	*Yes*	*Total*
Low MoCA at baseline	*No*	23,127 (24,272) n = 1157	30,231 (28,781) n = 53	23,438 (24,517) n = 1210
	*Yes*	24,060 (14,952) n = 84	33,548 (28,562) n = 133	29,875 (24,615) n = 217
	*Total*	23,190 (23,754) n = 1241	32,603 (28,586) n = 186	24,417 (24,632) n = 1427

## 4 DISCUSSION

### 4.1 Key results

In our main analyses (Table 3), having a low (bottom 10%) MoCA score at baseline was associated with 38% additional risk of subsequently becoming a high‐cost health care user. An equivalently low (bottom 10%) MMSE score at baseline was not significantly associated with additional risk of high health care costs after controlling for sociodemographics, clinical and functional factors, and prior health care use. The superior predictive power of MoCA over MMSE was also evidenced by the true positive rates: 40% of low‐MoCA participants became a high‐cost user within 8 years, compared to 35% of low‐MMSE participants. Mean costs are highest for the group who had low scores on both indices, next highest for those who had low MMSE only, then low MoCA only, and finally low scores on neither index.

### 4.2 Limitations

All variables in Table 1 and Table 2 are self‐reported, which increases the risk of biases related to response accuracy. Initial recruitment of community‐dwelling people means that ADRD diagnosis and declining cognitive function are more unusual in TILDA than in the general population of older people. A corollary of this sampling strategy, and a strength of our analyses, is that our cost data do not reflect high residential care expenditures, which may not be substantively avoidable, but rather hospital admissions, which may be avoidable or shortened with appropriate supportive care. Within the TILDA sample, health assessment participants who comprise our analytic sample were younger, more healthy and more socioeconomically advantaged than those who did not do the health assessment and are therefore excluded (Appendix). Non‐negligible measurement error has been noted previously on both cognitive scores.^34^ The cut‐points on MMSE and MoCA at the 10^th^ percentile were based on reason but ultimately pragmatic to ensure that any comparison was consistent on sample sizes; our results were substantively similar in sensitivity analyses with different cut‐points to the cognitive scores.

### 4.3 Interpretation

Prior literature on economics of cognition has noted that while the health care costs associated with ADRD are well established, much less is known about the costs of MCI.^35^, ^36^, ^37^ We analysed a sample of people who at baseline were over 50 years old and living in the community with <1% prevalence of self‐reported ADRD diagnosis (Table 1). An estimated 10%–20% of people aged 65+ will develop MCI, which is a significant risk factor for ADRD.^38^ The MoCA and MMSE indices were developed to identify differing populations: respectively, those living with MCI, who have lower costs than those with ADRD but are a much larger group^39^ and account disproportionately for health care costs at the population level; and those living with ADRD, who are approximately 1% of the population globally and have very high health care costs. These dynamics are borne out in Table 4, where a low MMSE score is better at predicting the very highest‐cost users (e.g. 98^th^ cost percentile up, among whom ADRD is prevalent), and a low MoCA score is better at identifying larger numbers of costly but not highest‐cost users (e.g. 90^th^‐98^th^ percentile).

True positive rates of 40% and 35% respectively are insufficient for screening patients for treatment pathways, but these statistics should be interpreted in the context of how challenging it is to identify prospectively those at risk of high costs. Prior efforts to combine diagnosis of serious illness, functional limitations and patterns of health care use into indices to predict high costs have reported sensitivity in a similar range; the so‐called “denominator challenge”.^6^

There's estimated to be more than 64,000 people are living in Ireland with dementia currently,^40^ approximately 1.3% of the population. Therefore by definition cognitive function scores will never be a powerful tool for prospectively identifying large proportions of the 10% of high‐cost users. However, as efforts to address the “denominator challenge” become more sophisticated, in recognition that high‐cost users are drawn from multiple potentially latent sub‐groups, people with lower cognitive function and at risk of cognitive decline will form one such important sub‐group. Moreover, as Ireland's young population ages, this sub‐group will increase in both total size and in mean per‐capita costs.

Our results highlight the scope for well‐known cognitive tests, and in particular MoCA, to provide a useful early indication that people are at risk for high costs. Future research should examine the scope to combine MoCA and MMSE, and potentially other measures, in the context of our results showing that the scores identify different parts of the cost distribution. Improved understanding of the linkage between future prevalence of specific conditions and their association with costs can also improve future predictions of health care expenditures.

## 5 CONCLUSION

Longitudinal follow‐up over eight years in a sample of people aged 50+ in Ireland found that MoCA was a more reliable predictor than MMSE of people subsequently becoming high‐cost health care users. Montreal Cognitive Assessment also had superior predictive accuracy to MMSE but the two scores identify somewhat different types of high‐cost user and so combining the approaches may improve predictive accuracy.

## Supporting information

Supplementary Material

## Data Availability

Replication of the results reported in this article requires access to the full TILDA dataset, which is held on secure servers at the study site at Trinity College Dublin (TCD). Researchers seeking access to the full TILDA dataset may apply to access the data on the TCD campus (tilda.tcd.ie); applications are considered on a case‐by‐case basis; all Stata do files and code employed in this paper will be made available to applicants on request.
