# Supplementary material for: Do Mini–Mental State Examination and Montreal Cognitive Assessment predict high‐cost health care users? A competing risks analysis in The Irish Longitudinal Study on Ageing
Source: Int J Geriatr Psychiatry. 2022 Jun 15;37(7):10.1002/gps.5766. doi: 10.1002/gps.5766 (PMC9328350; doi:10.1002/gps.5766)
Supplement: Supplementary file 1 — Supplementary Material [file GPS-37-0-s001.docx]

## Supplementary material

In this supplementary file, we provide baseline data on TILDA Wave 1 participants who did not complete the health assessment (and so are excluded from our analytic sample). We provide these data alongside the descriptors for the analytic sample (per Table 1 in the main document) in Appendix Table 1.

Those who did not complete the health assessment differed from the analytic sample in the following ways:

- Older (65.6 years on average versus 63.1 years)
- Higher prevalence of primary education as highest achieved (42.4% versus 26.0%)
- Higher prevalence of living alone (28.1% versus 20.0%)
- Lower prevalence of living in Dublin (17.9% versus 26.0%)
- Lower prevalence of private insurance (27.6% versus 45.2%)

Appendix Table 1 Descriptive data: characteristics of the analytic sample at baseline (Wave 1; N=8,173)

| Variable |  | **Analytic sample** | No health assessment |
| --- | --- | --- | --- |
|  |  | **n=5,856** | n=2,317 |
| **Age** | *Years* | **63.1 (9.3)** | 65.6 (10.6) |
| **Sex** | *Male* | **2687 (45.9)** | 1056 (45.6) |
| **Education** |  |  |  |
|  | *Primary/none* | **1523 (26.0)** | 981 (42.4) |
|  | *Secondary* | **2409 (41.2)** | 854 (36.9) |
|  | *Third/Higher* | **1922 (32.8)** | 480 (20.7) |
| **Living situation** |  |  |  |
|  | *Alone* | **1171 (20.0)** | 650 (28.10) |
|  | *With spouse* | **2384 (40.7)** | 857 (37.0) |
|  | *With others* | **2301 (39.3)** | 810 (35.0) |
| **Area** |  |  |  |
|  | *Dublin* | **1522 (26.0)** | 414 (17.9) |
|  | *Other urban* | **1607 (27.4)** | 706 (30.5) |
|  | *Rural* | **2727 (46.6)** | 1197 (51.7) |
| **Insurance**‡ |  |  |  |
|  | *Medical card* | **2110 (63.3)** | 1430 (61.7) |
|  | *Private insurance* | **2646 (45.2)** | 639 (27.6) |
|  | *Neither* | **596 (10.2)** | 247 (10.7) |
| **ADLs** | *Total /6* | **0.14 (0.55)** | 0.16 (0.66) |
| **IADLs** | *Total /6* | **0.12 (0.56)** | 0.21 (0.75) |
| **Cancer dx** | *Yes* | **361 (6.2)** | 151 (6.5) |
| **Heart dx** | *Yes* | **561 (9.6)** | 243 (10.5) |
| **ADRD dx** | *Yes* | **8 (0.1)** | 7 (.3) |
| **Multimorbidity** | *Yes* | **1466 (25.1)** | 603 (26.1) |

For binary and categorical variables: N (%). For continuous and count variables: Mean (St D). (I)ADL: (Instrumental) Activities of Daily Living. Dx = self-reported diagnosis. Cancer = excluding skin cancer. Heart = at least one of congestive heart failure, heart attack, stroke. ADRD: Alzheimer’s disease and related dementias. Multimorbidity = two or more of chronic conditions.
